# A case for considering individual variation in diel activity patterns

**DOI:** 10.1093/beheco/arx122

**Published:** 2017-09-11

**Authors:** Anne G Hertel, Jon E Swenson, Richard Bischof

**Affiliations:** a Faculty of Environmental Sciences and Natural Resource Management, Norwegian University of Life Sciences, NO-1430 Ås, Norway; b Norwegian Institute for Nature Research, NO-7485 Trondheim, Norway

**Keywords:** circadian activity, hunting risk, individual tactic, kernel density estimator, repeatability, *Ursus arctos*

## Abstract

There is a growing recognition of the role of individual variation in patterns emerging at higher levels of biological organization. Despite the importance of the temporal configuration of ecological processes and patterns, intraspecific individual variation in diel activity patterns is almost never accounted for in behavioral studies at the population level. We used individual-based monitoring data from 98 GPS-collared brown bears in Scandinavia to estimate diel activity patterns before the fall hunting season. We extracted 7 activity measures related to timing and regularity of activity from individual activity profiles. We then used multivariate analysis to test for the existence of distinct activity tactics and their environmental determinants, followed by generalized linear regression to estimate the extent of within-individual repeatability of activity tactics. We detected 4 distinct activity tactics, with a high degree of individual fidelity to a given tactic. Demographic factors, availability of key foraging habitat, and human disturbance were important determinants of activity tactics. Younger individuals and those with higher bear and road densities within their home range were more nocturnal and more likely to rest during the day. Good foraging habitat and increasing age led to more diurnal activity patterns and nocturnal resting periods. We did not find evidence of diel activity tactics influencing survival during the subsequent hunting season. We conclude that individual variation in activity deserves greater attention than it currently receives, as it may help account for individual heterogeneity in fitness and could facilitate within-population niche partitioning that can have population- or community-level consequences.

## Teaser

Animal behaviors are often described on the population level. Bears in Scandinavia, for example, are generally assumed to be active in the morning and afternoon. Using GPS-radio collar data from 98 brown bears, we show that bears, in fact, differ in their activity tactic. We illustrate 4 distinct tactics from strictly day active to strictly night active and bears that were measured over multiple years often used the same activity tactic.

## INTRODUCTION

Phenotypic plasticity allows for morphological or behavioral trait variation among individuals of one species, depending on environmental conditions or contexts (Komers 1997). Consistent individual variation, often termed an animal’s personality (Réale et al. 2007), may thereby limit its range of behavioral plasticity (Dingemanse et al. 2010). This leads to a spectrum of different behavioral phenotypes or tactics within populations. In migratory species, for example, some individuals may depart toward breeding grounds earlier than others (Duriez et al. 2009) or apply a different tactic when trying to maximize intake of newly emerging, highly nutritious vegetation (Bischof et al. 2012). Behavioral strategies that are beneficial for some aspects of life may, however, simultaneously be disadvantageous for others (Stamps 2007). For example, selection for open habitats (Lone et al. 2015) and higher movement rates (Ciuti et al. 2012a) may increase food intake, which can translate into larger body sizes and higher reproductive success, but may also increase the probability of being detected by a predator. Individual behavioral variation is therefore beneficial and important at the population level because an assortment of different strategies makes a population more adaptable to changing conditions (Wolf and Weissing 2012; Dingemanse and Wolf 2013).

Activity is one fundamental attribute of animal behavior (Réale et al. 2007) and is often concentrated at certain times of the day, known as diel activity. Diel activity is primarily governed by encounter probability with food (Klinka and Reimchen 2009), and predators (Lima and Dill 1990; Alós et al. 2012), thermoregulation (Maloney et al. 2005), and the dark-light regime (Ensing et al. 2014; Heurich et al. 2014). Sympatric species may avoid each other temporally, thereby leading to niche partitioning (Case and Gilpin 1974) and species coexistence (Gerber et al. 2012). For example, in preferred habitats, cheetahs (*Acinonyx jubatus*) avoid lions (*Panthera leo*) at fine temporal scales, thereby securing access to resources while avoiding interference competition (Swanson et al. 2016). In predator–prey systems, diel activity drives interactions (Monterroso et al. 2013), thereby shaping food intake of predators and mortality risk for prey. Similarly, hunted species may react to human disturbance with shifts of activity to avoid humans temporally (Brook et al. 2012; Ciuti et al. 2012b; Marchand et al. 2014). This can have far-reaching ecosystem consequences. For example, Brook et al. (2012) demonstrated that a top predator, the dingo (*Canis lupus dingo*), shifted its activity away from human activity on properties where it was hunted. This in turn leads to a release of activity by a mesopredator, the feral cat, which resulted in higher predation rates on prey species. However, few studies have tested whether activity shifts and differences in individual activity indeed increase an individual’s survival under predation pressure (but see Pizzatto et al. 2008; Ciuti et al. 2012a; Lone et al. 2016). Lastly, animals inhabiting areas with more human-built infrastructures, like roads (Ordiz et al. 2014) or wildlife crossings (Barrueto et al. 2014) may display different diel activity patterns as then their conspecifics inhabiting more remote areas. When species shift diel activity asynchronously in response to infrastructures (Barrueto et al. 2014), they have the potential to alter species interactions at the community level. Despite the demonstrated importance of diel activity on fundamental ecological processes and increasingly large amounts of individual-based monitoring data collected by many wildlife research projects, individual differences in activity tactics at the population level, their drivers, and their ecological consequences are still rarely quantified.

Our objectives were to 1) quantify individual variation in activity patterns and test for the presence of distinct diel activity strategies, 2) to identify extrinsic and intrinsic determinants of tactic expression, 3) determine whether activity tactics have consequences for immediate survival, and 4) test for within-individual repeatability of activity tactics for individuals that were monitored over several years.

Our model species, the Scandinavian brown bear (*Ursus arctos*) is generally thought to follow a bimodal activity pattern, with activity occurring in the early morning and afternoon hours and resting during midday and night (Moe et al. 2007). There is, however, evidence for within-population variation in diel activity. For example, female brown bears are more diurnal than males (Ordiz et al. 2007), particularly when they are accompanied by cubs, which are vulnerable to infanticide by male bears (Steyaert et al. 2013). Bears further avoid humans by becoming more nocturnal at the onset of the hunting season (Ordiz et al. 2012) and after encounters with humans (Ordiz et al. 2013), and decrease foraging activity when mortality risk is highest (Hertel et al. 2016b). It remains unknown whether bears with reduced activity during hours of high mortality risk have a higher likelihood of survival than individuals that remain active at those times. It has been shown that bears of different demographic groups and after disturbance events exhibit behavioral flexibility in their diel activity pattern, and it is, therefore, conceivable that activity tactics may also vary among individuals per se. We utilize a method that was originally developed to estimate the overlap of activity patterns between species recorded with trail cameras (Meredith and Ridout 2014) to quantify individual activity profiles of bears derived from high-resolution GPS movement data.

## METHODS

### Study area

The study area was situated in southcentral Sweden (61°N, 14°E). The area is comprised of intensely managed boreal forest, interspersed by lakes and bogs. Scots pine (*Pinus sylvestris*) is the dominating tree species, followed by Norway spruce (*Picea abies*). Human population density in the study area is low (4–7 inhabitants/km^2^: Ordiz et al. 2014). An intense network of forest roads (0.7 km/km^2^: Martin et al. 2010), however, facilitates easy access into the study area. Recreational activities in the forest are mainly concentrated in the summer and autumn months (Ordiz et al. 2011).

### Bear data

Bears were darted from a helicopter and equipped with GPS-GSM neck collars (Vectronic Aerospace GmBh, Berlin, Germany) and a VHF transmitter implant (IMP 400L; Telonics, Mesa, AZ), see Arnemo and Fahlman (2011) for details on capture and handling. All animal capture and handling were approved by the Ethical Committee on Animal Experiments in Uppsala, Sweden and the Swedish Environmental Protection Agency (Uppsala Djurförsöksetiska Nämd permissions C59/6, C47/9 and C7/12).

We used GPS relocation data of brown bears taken at 30-min intervals over a 3-week period immediately before the fall hunting season, from 1 August until 20 August 2007–2013. The GPS data were collected into the Wireless Remote Animal Monitoring (Dettki et al. 2014) database system for data validation and management. Locations were cleaned for dilution of precision (DOP) values >10 and experimental approaches by humans on foot (Moen et al. 2012). Experimental approaches have a pronounced effect on regular activity patterns for 72 h after the disturbance (Ordiz et al. 2013), we therefore also excluded all locations during the 3 days following an approach. We used positions of solitary bears (i.e., females were not accompanied by cubs) that were >2 years of age with at least 350 active relocations. Our activity classification was strictly movement based. We calculated the straight-line distance between bear positions and classified locations as active when the movement distance exceeded 25 m (Ordiz et al. 2011; Hertel et al. 2016b, a sensitivity analysis for the 25 m cutoff value is provided in Supplementary Material 1).

### Approximating the activity distribution

**Figure 1:**
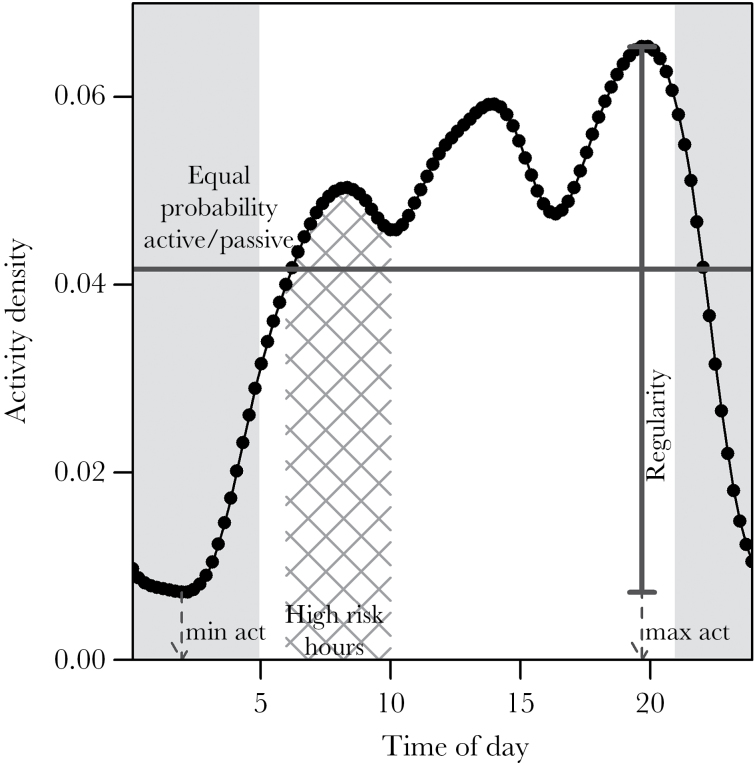
An example of the Kernel density distribution of activity shown for one brown bear in south-central Sweden. Time of minimum/maximum activity and activity regularity are indicated with arrows. Hours with high mortality risk during the hunting season (06:00–10:00) are highlighted with hatched lines. The horizontal line marks the activity density at which any individual has an equal likelihood of being detected as active or not active. Night hours (21:00–5:00) are shown by gray shading.

Using the function densityPlot from the “overlap” package (Meredith and Ridout 2014), we fitted kernel density curves to the timing of circadian active behavior for each individual bear. We extracted the *x* (time in radians) and *y* (activity density) coordinates underlying the densityPlot, which returns the smoothed activity density between 21:00 on day *t* − 1 and 3:00 on day *t* + 1, where estimates between 21:00 and 0:00 on day *t* − 1 are equal to estimates between 21:00 and 0:00 on day *t*. We truncated the time window to the 24-h cycle which yielded a total of 102 density estimates. Since a day is a periodic event, the first density estimate is a continuation of the last density estimate. Density curves calculate the relative occurrence of an individual’s active observations over the 24-h cycle, thereby accounting for differential sampling effort among individuals. As activity within a day is a discrete behavior (active vs. passive) sampled over the course of several days, the density smoother should be interpreted as the relative probability of being active (Figure 1).

### Quantifying individual activity measures

**Figure 2:**
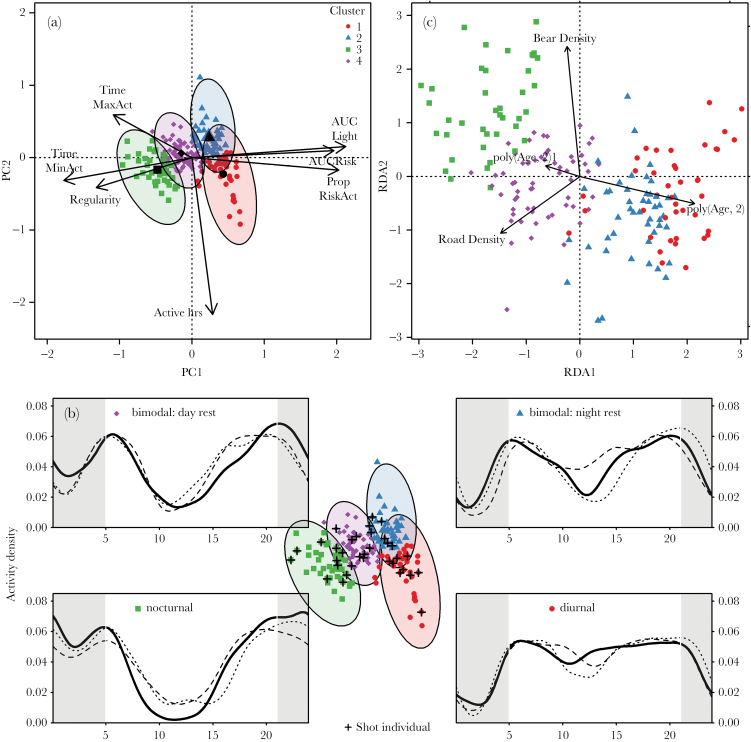
Placement of individual activity patterns along 7 activity measures—time of minimum (TimeMinAct) and maximum activity (TimeMaxAct), the deviation between the two as a measure of regularity in the activity pattern (Regularity), activity during risky hours (AUC Risk) and light hours (AUC Light), and the proportion of the high risk hours (PropRiskAct) and the proportion of the day (Active hours) an individual was more likely to be active than not active—presented in 2-dimensional space along the first 2 PCA axes (a). Clustering of activity patterns (along the first 2 PCA axes), and their cluster centroids (black symbols) are shown in panel (a). Associations of clusters with their respective activity tactics are shown in panel (b). Subplots in panel (b) show the kernel density distribution of activity for 3 representative activity profiles in each of the 4 activity tactics. Night hours (21:00–5:00) are shown by gray shading. Panel (c) shows the association of activity phenotypes with an individual’s age, proportion of good foraging habitat, road density, and bear density in their home range. Individuals that were killed in the subsequent hunting season are marked with a cross in panel (b).

We extracted the time of day at which an individual’s density of activity was lowest and highest, that is, when an individual was most often active or inactive and calculated the difference between the highest and lowest density values as an indicator of the regularity of activity behavior. A low regularity index is indicative of a less pronounced activity rhythm and activity at different times of day across the study period, whereas a high regularity indicates a low variance of the activity pattern. We quantified how active an individual was during hours of high mortality risk (6:00–10:00 (Hertel et al. 2016b)) by calculating 1) the area under the curve (AUC) during hours of high risk (hatched area—Figure 1) and 2) the proportion of high-risk hours in which an individual was more likely to be active than not active (density coefficient > 0.0465, horizontal gray line—Figure 1). Likewise, we calculated how active an individual was during light hours (after sunrise at 5:00, before sunset at 21:00) using AUC (nonshaded AUC—Figure 1) and the proportion of the day an individual was more likely to be active than not active.

### Clustering of activity measures

We used principal component analysis (PCA) on the 7 scaled activity measures (time of minimum and maximum activity, regularity, AUCRisk, AUCLight, proportion of time active under risk, cumulative active time), to identify patterns in the activity measures across individuals. We used k-means cluster analysis on the PCA scores of the first 2 axes to group individuals into distinct activity tactics. We inspected sum of squared error scree plots to determine the optimal number of clusters. After choosing the most parsimonious number of clusters, individuals were then assigned to the cluster with the closest centroid to their position along the 2 PCA axes.

### Within-individual repeatability of activity tactic

We selected all individuals that were monitored in more than 1 year to evaluate whether animals were more likely than expected to be assigned into the same cluster and thus express the same activity tactic across multiple years. For each individual, we determined the most prevalent of its expressed tactics as the focal tactic. All observations of an individual were then classified as belonging to the focal tactic or to any of the other tactics. For individuals with 2 or more equally common tactics, one was randomly designed as focal. For each individual with a given number of monitoring years, we simulated the random assignment into focal or nonfocal tactics, given the number of available tactics. Using logistic regression, we analyzed whether the probability that an observation belonged to the focal tactic was higher in the observed data than in the simulated data.

### Covariate effects on activity patterns and consequences for survival

Next, we used redundancy analysis (RDA), a constrained ordination method, to identify environmental determinants explaining the observed patterns in activity. The variance explained by each environmental variable is partitioned along the RDA’s ordination axes. In the ordination plot, the environmental variables for which variance is mostly reflected by the first axis are shown as a parallel arrow along the *x* axis, with the arrow length indicating the strength of the effect. An individual’s position in the 2-dimensional space (if 2 axes are displayed) depends accordingly on its associated value for the environmental constraints. We fitted a set of intrinsic (sex and age), environmental (bear population density and proportion of berry habitat), and disturbance measures (road density, annual number of captures, and experimental disturbances; Støen et al. 2010; Moen et al. 2012), survival in the upcoming hunting season starting 21st of August) onto the activity data. Road density was obtained from the Swedish National Road Database (NVDB www.trafiksverket.se; license ID: i2014/00764). Habitat in the Swedish CORINE Landcover map (Svenska CORINE Marktäckedata) was reclassified as berry habitat or no berry habitat, depending on its probability of berry occurrence (Hertel et al., 2016a). Forested habitats were classified as berry habitat, lakes, bogs, and pastures as no berry habitat. Bear density was estimated from DNA sampling of country wide collected scat samples and observations of bears during the fall moose (*Alces alces*) hunting season, initiated by the Swedish Association for Hunting and Wildlife Management (Kindberg et al. 2011). Bear density was estimated on an annual basis with a resolution of 10 km × 10 km (Leclerc et al. 2017; Frank SC, Leclerc M, Pelletier F, Rosell F, Swenson J, Bischof R, Kindberg J, Eiken HG, Hagen S, Zedrosser A, unpublished data) following the method of Jerina et al. (2013). Bear density, the proportion of berry habitat, and road density were extracted within the individual’s autumn 95% kernel home range, which was constructed from all active and inactive relocations during the study period. Age was included as a second-order polynomial, and we tested for an interaction between age and sex. We used an automated model selection procedure, the ordistep function in the vegan package (Oksanen et al. 2016), with 200 permutations to find the independent parameters that best explained the placement of individuals along the PCA axes. We also tested whether a bear’s survival was independent of its diel activity tactic using a chi-squared test.

## RESULTS

Data from 98 individuals (73 females, 25 males) representing 196 bear years, comprising a total of 183.973 relocation intervals were included in the analysis. Using a cutoff value of 25 m, 68% (125.666) positions were categorized as active, and 32% (58.307) positions were categorized as not active. The number of active positions per bear year from which individual activity profiles were constructed ranged between 353 and 705. Thirty-five individuals were killed during the subsequent hunting season.

### Individual activity patterns

Multivariate PCA and cluster analysis revealed 4 distinct activity patterns (Figure 2a). There was a drop in the within-group sum of squares after 4–5 clusters (Figure 1 in Supplementary Material 2), but an examination of the clustering (Figure 2 in Supplementary Material 2) and inspection of activity profiles in the different clusters suggested that 4 clusters more appropriately reflected activity tactics in our population. Clustering explained 72.7% of the observed variation. The distribution of bear years into the 4 clusters was approximately even with 42 bears (21%) in cluster 1, 48 (25%) in cluster 2, 44 (22%) in cluster 3, and 62 (32%) in cluster 4.

The first 2 axes of the PCA explained 65% of the variation in the data (PC1 50%, PC2 15%, PC3 12%, and PC4 12%). Activity during risky and light hours was strongly reflected by positioning along the first PCA axis. Individuals in clusters 1 and 2 were thus more exposed to risky hours than individuals in clusters 3 and 4 (Figure 2a). The proportion of active hours was associated with the second axis, indicating that individuals in clusters 1 and 3 were more likely to be active than not active during a larger proportion of the day than those in clusters 2 and 4. The timing of minimum activity was associated with the first axis; 106 individuals preferably rested during midday (clusters 3 and 4), whereas 90 individuals rested most consistently during the night (clusters 1 and 2). Activity profiles of individuals in clusters 3 and 4 were more consistent than for those in clusters 1 and 2, which was reflected by the first and third axes (see Figure 3 in Supplementary Material 2 for placement of individuals along the first 3 PCA axes). Likewise, the timing of maximum activity was reflected by the first and third axes, with most individuals (79%) being active later in the day, but individuals in cluster 1 tended to have their peak activity in the morning. Individuals in cluster 3 were generally more nocturnal and individuals in cluster 1 more diurnal (Figure 2b). Variation in activity measures of bimodally active individuals was partitioned into 2 clusters, ones that rested primarily during the day (cluster 4), and ones that rested primarily during the night (cluster 2, Figure 2b). Individuals were categorized into phenotypes according to their closest cluster centroid (Figure 2a), individuals in overlapping areas of cluster polygons are therefore similar to each other, despite being categorized into different clusters. Overlap occurred in particular between the diurnal and bimodal with preferred night rest tactics (clusters 1 and 2, Figure 2a), indicating that categorization into one or the other phenotype must be considered with caution for individuals falling into the overlapping area.

### Within-individual repeatability of activity patterns

**Figure 3:**
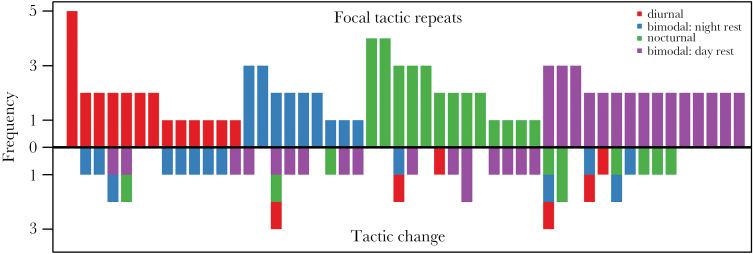
Within-individual repeatability of activity tactics. For individuals that were observed in 2 or more years, the focal tactic was set to the activity pattern most often applied by this individual. Bars above the zero line present the number of years in which an individual applied its most common tactic (color coded by activity tactic). Bars below the zero line represent years in which an individual applied a tactic other than its focal tactic.

For 50 individuals, activity tactics were obtained for multiple years (148 bear years). The number of observations per individual ranged from 2 to 6 years (mean ± SD = 2.96 ± 1.07). For each individual’s number of observations, the distribution into focal and nonfocal tactic under random assignment was simulated 10 times, yielding a total of 1480 random observations. Bears selected their focal tactic significantly more often than expected by chance (β ± SE = 1.368 ± 0.186, *z* = 7.369, *P* < 0.001) and 60% of bears used one tactic more often than any other tactic (Figure 3).

### Covariate effects on activity patterns and consequences for survival

The global model including 7 individual-based variables performed significantly better than the intercept only model (df = 10, *F*-test = 2.132, number permutations = 999, *P* = 0.002). The best model included the effect of age (df = 2, *F* = 2.86, *P* = 0.017), bear population density (df = 1, *F* = 4.64, *P* = 0.007), and road density (df = 1, *F* = 4.062, *P* = 0.008). Total explained variation by the constraining variables was low, however (*R*^2^ = 0.07, adj. *R*^2^ = 0.051, Table 1) with most variation explained by the unconstrained PC axes (Table 1). The accumulated explained variation by the first 2 RDA axes was 94%.

**Table 1:** Scores for important constraining predictor variables onto the first 4 RDA axes

	RDA1	RDA2	RDA3	RDA4
poly(Age,2)1	−0.228	0.071	−0.188	0.953
poly(Age,2)2	0.764	−0.181	−0.615	0.075
BearDensity	−0.086	0.866	−0.487	−0.071
RoadDensity	−0.528	−0.379	−0.655	−0.386
Proportion explained variation by constrained axes	0.038	0.028	0.003	0.002
Proportion explained variation by unconstrained axes	0.470	0.140	0.119	0.097
Accumulated explained variation	0.54	0.4	0.04	0.02

Proportion of variation explained by the constrained an unconstrained axes and accumulated variation explained by each axis.

Older bears were more diurnal and rested primarily during the night (Figure 2c), whereas younger bears were more nocturnal. High bear density was reflected along the second axis (Table 1) and associated with the nocturnal activity tactic. Higher road densities were associated with preferred day resting and activity during night. Nevertheless, nocturnal or diurnal activity tactics were not associated with survival in the subsequent hunting season (df = 1, *F* = 0.9, *P* = 0.4). Activity tactics did not differ between hunter-killed and surviving individuals (Figure 2b, χ^2^ = 1.387, df = 3, *P* = 0.709).

## DISCUSSION

We detected pronounced individual variation in diel activity along a gradient from strictly nocturnal to strictly diurnal activity within our study population. Cluster analysis categorized activity patterns into 4 distinct activity tactics (Figure 2b), mainly structured by timing of principal activity and resting, which occurred in approximately equal frequency. We also found that individual bears were likely to repeat the same tactic over multiple years (Figure 3). Activity tactics were influenced by a series of individual and environment attributes (Figure 2c). Survival in the upcoming hunting season was not affected by the activity tactic that an individual used prehunting.

Bears responded to increasing human access into their home range by being active primarily during the dark hours, most likely to avoid humans temporally, which is consistent with previous findings for our study population (Ordiz et al. 2014) and elsewhere (Stillfried et al. 2015). Individual bears responded likewise to increasing bear density. Intraspecific temporal niche partitioning has been described for grizzly bears in the Greater Yellowstone Ecosystem, where females are primarily diurnal, whereas males are nocturnal (Schwartz et al. 2010). In our population, younger individuals were more nocturnal than older ones, indicating a temporal niche partitioning driven by social organization (Bergmüller and Taborsky 2010). Because bears seem to prefer foraging during the daylight hours (MacHutchon et al. 1998) and foraging is their main activity in summer, high human or bear density seem to temporally displace individuals, particularly younger ones, to nonpreferred foraging times. We did not detect a sex effect on the propensity to be more diurnal or nocturnal. Importantly, the number of experimental disturbances (Moen et al. 2012) and repeated captures of individuals did not seem to affect baseline activity tactics beyond the previously demonstrated effect on movement patterns after the disturbance event (Ordiz et al. 2013); data of which were excluded from the analysis.

Active behavior may increase an individual’s probability to be killed during the hunting season if activity increases detectability, as has been suggested, for example, roe deer (*Capreolus capreolus*) (Lone et al. 2016) and red deer (*Cervus elaphus*) (Ciuti et al. 2012a). Contrary to our expectations, however, we did not find that an individual’s activity tactic affected its probability to survive the hunting season. We explain this apparent lack of harvest selectivity for phenotypes that are more active during the hours of highest mortality risk in the morning (Hertel et al. 2016b) with the hunting method used for bear hunting in Sweden. In recent years, bears are hunted almost exclusively with the use of scent-motivated hunting dogs that search actively for bears (Bischof et al. 2008; Swenson et al. 2017). Hunters typically drive along forest roads until they find signs of a bear that recently had crossed the road, like a fresh scat, and then release the dog, which follows the scent track of the bear. For mortality risk to be increased by activity, movement must increase detectability, which we do not expect happens with this hunting method. A limitation of our line of evidence is, however, that we linked survival to activity tactic before the onset of the hunting season, not during the hunting season itself. This was because 19 of the 35 hunter-killed bears in our study were killed in the first week of the hunting season (7 on the first day). Activity profiles built from very few observations are less reliable and would not be comparable among individuals that were removed before the end of the sampling period (right censored data). Bears may, however, sense changes in predation risk with onset of the hunting season and alter their movement pattern accordingly, as has been documented previously (Ordiz et al. 2012; Hertel et al. 2016b). Apart from hunting mortality, diel activity may affect other constituents of fitness that we did not test, for example, number of male–female encounters during the spring mating season, which affects reproductive success for males and survival of cubs accompanying their mothers (Steyaert et al. 2013).

Individual bears were likely to apply the same activity tactic when monitored over several years and all 4 activity tactics occurred as a focal tactic (i.e., the tactic most used by an individual). The magnitude of activity is regularly used as 1 of 5 behavioral traits to assess animal personality (Réale et al. 2007) and is often correlated with an individual’s degree of boldness (Sih et al. 2004). We argue that under natural conditions, not only magnitude of activity but also its temporal distribution, that is, diel activity tactic, could be correlated with boldness of an individual. Specifically, more diurnal individuals are active under preferred foraging times (MacHutchon et al. 1998) while accepting a higher risk of human encounters which hints toward a bolder personality type than more nocturnal individuals. To substantiate that diel activity indeed reflects consistent individual trait variation, one should evaluate within-individual correlation of diel activity across life stages or seasons when different needs and constraints govern a bear’s behavior, such as mating (Steyaert et al. 2012), cub protection (Steyaert et al. 2013), or dispersal (Støen et al. 2006). Further evidence for consistent individual variation in our bear population comes from their selection for bog and clearcut habitats (Leclerc et al. 2016) and it would be interesting to see whether individual variation is correlated across behavioral domains.

Our analytical approach used multiple measures quantified from activity density curves determined from movement between GPS relocations. A limitation of a strictly movement based activity classification is that spatially restricted active behaviors, like for example, foraging at a carcass site (Rauset et al. 2012) may erroneously be classified as inactivity. Our study period was however placed in the autumn when bears in Sweden forage almost exclusively on berries, a behavior which can well be identified from GPS relocations by its continuous short distance movements (Hertel et al. 2016a). Our result that 4 distinct activity strategies exist in our population persisted when reducing (15 m) or increasing (37.5 m, 50 m) the cutoff value for activity underlining the robustness of our findings (refer also to Supplementary Material 1). The distribution of individuals classified into each tactic changed in an ecologically reasonable manner, that is, fewer individuals categorized as diurnal with increasing cutoff values because bears move shorter distances during midday (Ordiz et al. 2012). Ecological knowledge of season and forage specific movement patterns, a priori data exploration of the distribution of movement distances, and post hoc sensitivity analysis using a range of activity cutoff values can assist choosing an appropriate cutoff distance and to assess the robustness of results. Active behavior can also be determined from dual- or tri-axial accelerometers (Gervasi et al. 2006; Shepard et al. 2008). This rapidly developing technology allows identification of behaviors from acceleration patterns. Because our method is not restricted to quantifying activity alone, it could also be used to construct diel curves for specific behaviors. Moreover, our approach is not limited to patterns within the 24-h cycle, but can be used to describe among-individual differences in any behavior that can be expressed as intensity or proportional occurrence over a temporal trajectory. Because Kernel density curves reflect the relative temporal distribution of activity over a set time period, the sum and mean of density estimates is identical for all individuals. Density curves can thus be used to compare temporal patterns among individuals with unequal sample sizes or incomplete detection, which are common in GPS relocation datasets. We know that cumulative activity may differ between sexes, with age, or reproductive class (Garshelis et al. 1983; Steyaert et al. 2014); the approach used here would have to be adjusted to allow for comparisons of the absolute magnitude of activity (i.e., between individuals that spend more or less time active).

For statistical purposes alone, individual differences should be taken into consideration more regularly, especially when models presume a certain data distribution. For example, activity is often analyzed using additive models and controlling for individual variation with a random intercept (Heurich et al. 2014; Zuur et al. 2014). However, a random intercept does not control for differences in the shape of the smoother (analogous to the slope in linear regression). When individuals that are active at different times of day are entered into the same analysis, their differential data distribution thereby violates the underlying model assumptions and potentially influences conclusions drawn from the model output.

The key role that species-specific diel behavior plays in structuring communities, for example by determining interactions between predators and prey (Brook et al. 2012; Monterroso et al. 2013) or intraguild temporal niche partitioning (Valeix et al. 2007; Schwartz et al. 2010; Swanson et al. 2016), is undisputable. Intrapopulation individual variation in diel behavior of wildlife is most commonly described for different demographic groups (Schwartz et al. 2010; Steyaert et al. 2013) or when animals are exposed to differential environmental conditions (Barrueto et al. 2014; Ensing et al. 2014; Heurich et al. 2014) and an individual’s diel activity tactic may thereby affect the role it exerts within its multispecies community. For example, when predation success is highest during distinct periods of the day, but predators vary in their diel activity tactic, temporal access to prey, foraging strategy, and diet composition may consequently vary between individuals (Estes et al. 2003; Araújo et al. 2011). That individuals contribute differentially to the dynamics of this predator–prey relationship has broad implications for community ecology (Bolnick et al. 2011).

The concepts behavioral plasticity, personality, and reaction norms have been mainly studied in short-lived nonmammalian species, particularly in controlled environments (e.g., table 1 in Bergmüller and Taborsky 2010; Biro and Stamps 2008; Dingemanse et al. 2010). Individual variation of activity specifically may cause variation in reproductive success, when activity has a significant and positive effect on food intake (Biro and Stamps 2008) and therefore may explain individual variation in fitness. However, few studies on large mammals (Müller and von Keyserlingk 2006) and, to our knowledge, none on free-living wildlife have explicitly tested this. This is particularly relevant, because of the importance of adaptive behavioral strategies on life histories and population persistence, especially in species with long generation times (Refsnider and Janzen 2012).

## SUPPLEMENTARY MATERIAL

Supplementary data are available at *Behavioral Ecology* online.

## FUNDING

This work was supported by the Swedish Environmental Protection Agency, Norwegian Directorate for Nature Management, Swedish Association for Hunting and Wildlife Management, the Austrian Science Fund, and the Research Council of Norway. The funders had no role in designing the study, or preparation of the article.

## Supplementary Material

Supplementary Material 1

Supplementary Material 2
