# Supplementary material for: A case for considering individual variation in diel activity patterns
Source: Behav Ecol. 2017 Sep 11;28(6):1524–31. doi: 10.1093/beheco/arx122 (PMC5873257; doi:10.1093/beheco/arx122)
Supplement: Supplementary Material 1 [file arx122_suppl_supplementary-material1.docx]

**Title:** A case for considering individual variation in diel activity patterns.

**Journal:** Behavioral Ecology

**Authors:** Anne G. Hertel, Jon E. Swenson, Richard Bischof

**Corresponding Author E-mail**: [anne.hertel@nmbu.no](mailto:anne.hertel@nmbu.no)

**Supplementary material 1**

**Sensitivity analysis of activity cutoff value**

In the main body of our manuscript we used a cutoff of 25 meter distance between relocations to discern activity from movement data. We assumed that an individual which moved more than 25 m over a 30-minute interval could be considered active, whereas an individual that moved up to 25 meters could be considered stationary and thus inactive. Bears usually rest (i.e. are inactive) in dense vegetation (Ordiz et al., 2011), where accuracy of GPS locations is lower compared to more open habitats. The increase in position error leads to greater distances between relocations even though the bear is indeed stationary, which could lead to biased activity designations. Here we provide an analysis of how robust the clustering of bears into four distinct activity tactics, re is towards changes in the cutoff (Fig. 1) value to categorize positions as active or non-active.

**Figure 1:**

Distribution of all relocation intervals (n = 183.973) with a given movement distance. The x axis is truncated to 2000 m movement distance, longer movements were extremely rare (< 0.1%). The peak between 5 and 20 m represents stationary behavior which in our study is referred to as non-active behavior. The inset shows an enlargement of the data distribution between 0 and 100 m movement distance. The solid line at 25 m represents the activity cutoff used in the main body of the manuscript, the dashed lines activity cutoffs at 15, 37.5 and 50 m movement distance.


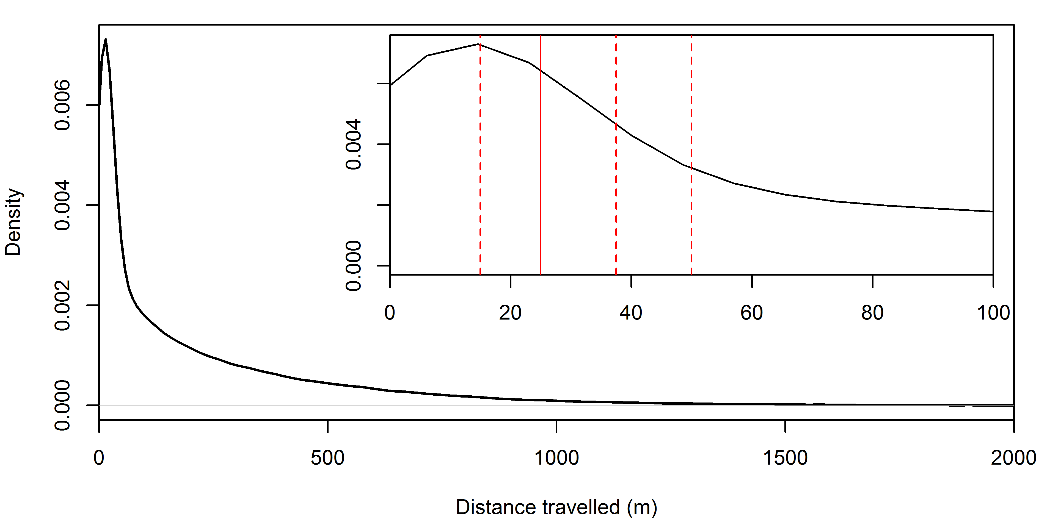


Using ordination analysis the four tactics persisted when the activity cutoff value was either increased (37.5 or 50 m) or decreased (15 m). Sum of squared error scree plots (Fig. 2) for all three alternative cutoffs were similar to the one obtained from the 25 m cutoff and suggested an optimal number of clusters of 4-6.

Association of activity measures with axis 1 and 2 were equal for all four cutoff values (Fig. 3 & 4). Using four clusters, the number of individuals categorized into either one cluster/tactic (Table 1) changed as we increased the cutoff from 15 m to 50 m. Specifically, the number of individuals categorized as diurnal decreased, bimodal: nightrest increased and bimodal: dayrest decreased. This is not unexpected, given that most bears generally move shorter distances during midday (Ordiz et al., 2012).

Increasing the cutoff value for activity beyond 25 m would increase the error of miss-classifying spatially restricted behaviors as inactivity. The study took place during the berry season and berry foraging in bears is characterized by slow sinuous movements (Welch et al., 1997).

Stationary behavior can be delineated with relatively high accuracy when examining the density distribution of distance travelled between relocations (Fig. 1). This distribution peaks around 5 – 20 m. Bears primarily rest in very dense vegetation with much cover where GPS relocation errors are expected to be highest. We therefore also argue that decreasing the cutoff down to 15 meters would miss-classify more non-active positions as active.

Choosing a cutoff value should be based on a combination of ecological knowledge, data exploration and the robustness of results. The changes we observe in our results (i.e. decreasing number of individuals categorized as diurnal with increasing activity cutoff) can be explained ecologically because bears move shorter distances during midday.

**Table 1:**

Number of individuals categorized into each of the four activity tactics depending on the activity cutoff value used. The in the manuscript used cutoff value of 25 m is represented in bold font.

|  | Activity cutoff | | | |
| --- | --- | --- | --- | --- |
|  | 15 m | **25 m** | 37.5 m | 50 m |
| diurnal | 46 | **42** | 37 | 28 |
| bimodal: nightrest | 39 | **48** | 66 | 70 |
| bimodal: dayrest | 66 | **62** | 71 | 55 |
| nocturnal | 45 | **44** | 22 | 43 |

**Figure 2:**

Sum of squared error scree plots to determine the optimal number of activity-tactic clusters from the placement of activity patterns along the first two PCA axes using acticity cutoff values of 15, 37.5 and 50 m. The within-group sums of squares ceased to decrease strongly after 4 to 6 clusters in all cases, indicating that clustering into more subgroups did not improve the variation explained by the clustering.


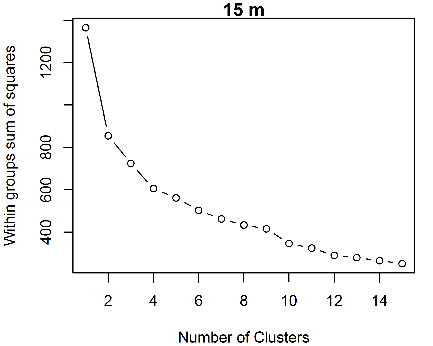

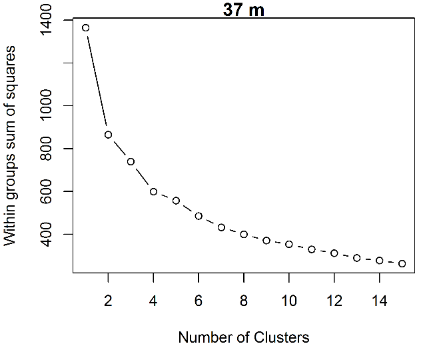

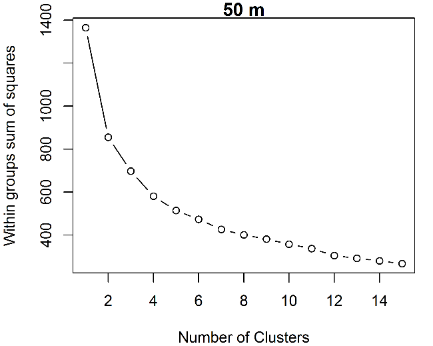


**Figure 3:**

Increasing the activity cutoff value from 25 m to 37.5 m (left) or 50 m (right).


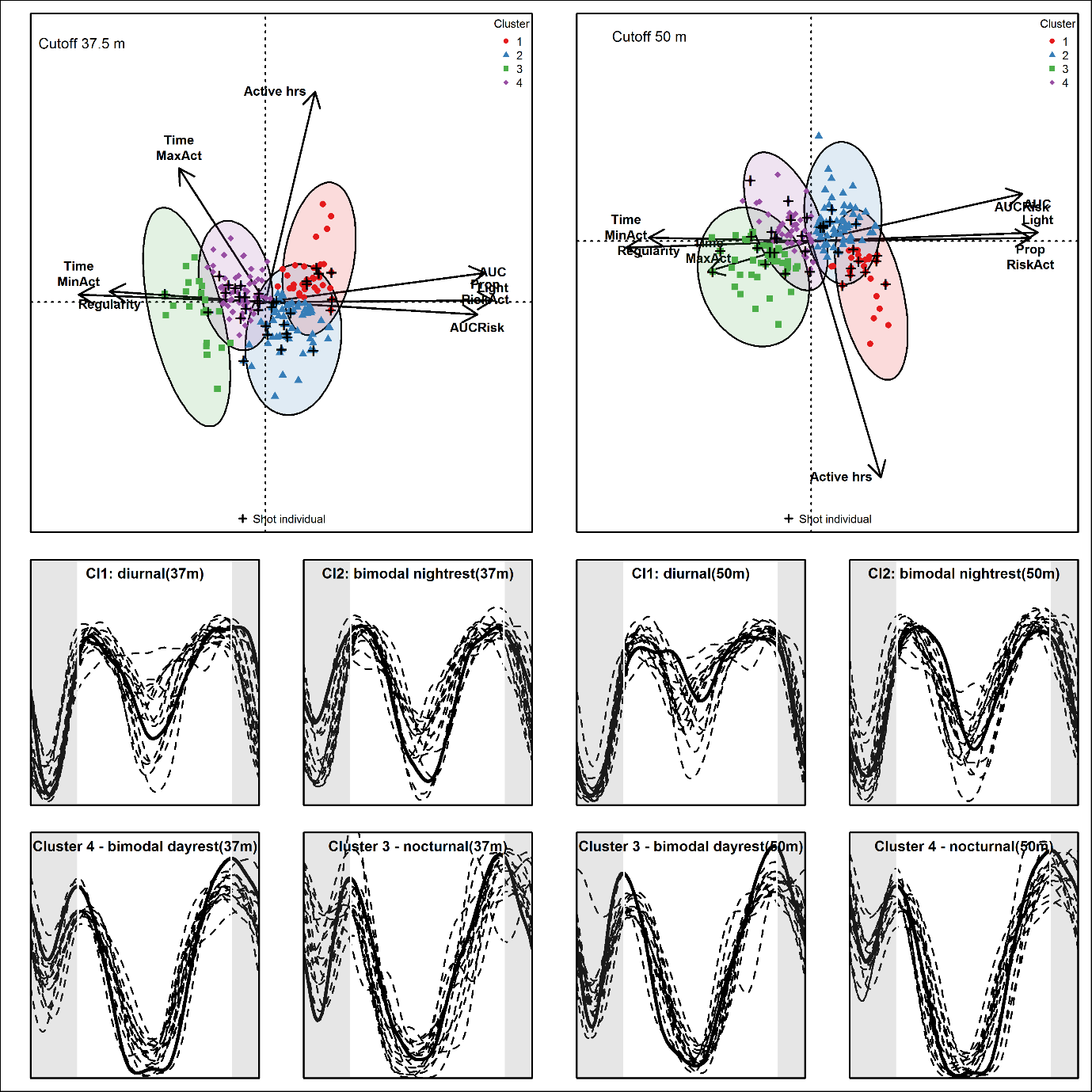


**Figure 4:**

Decreasing the activity cutoff value from 25 m to 15 m:


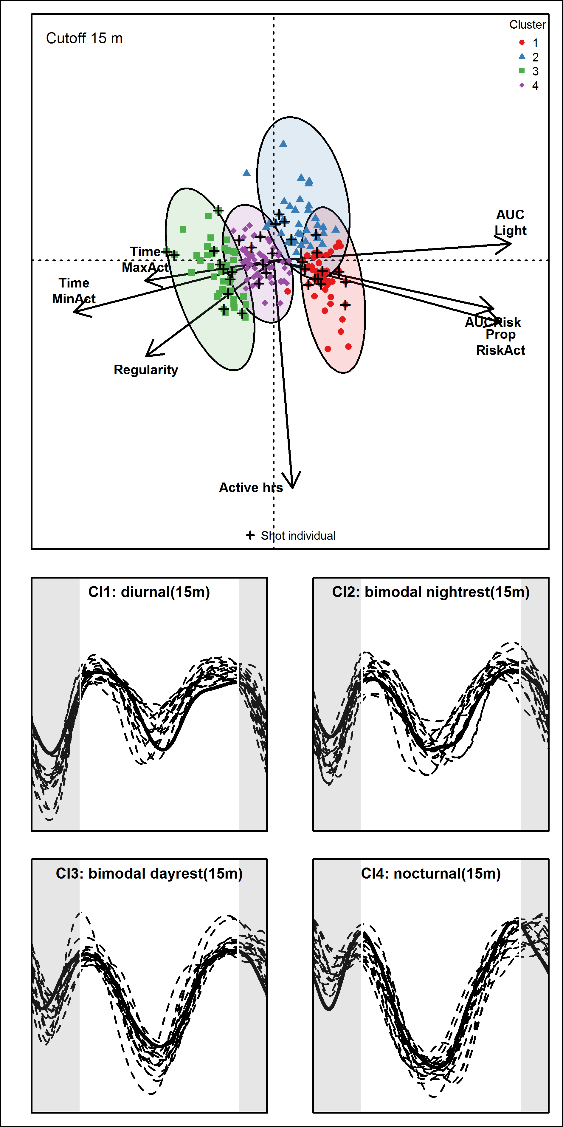


Ordiz A, Stoen O-G, Delibes M, Swenson JE, 2011. Predators or prey? Spatio-temporal discrimination of human-derived risk by brown bears. Oecologia 166:59-67. doi: 10.1007/s00442-011-1920-5.

Ordiz A, Støen O-G, Sæbø S, Kindberg J, Delibes M, Swenson JE, 2012. Do bears know they are being hunted? Biological Conservation 152:21-28.

Welch CA, Keay J, Kendall KC, Robbins CT, 1997. Constraints on frugivory by bears. Ecology 78:1105-1119. doi: 10.1890/0012-9658(1997)078[1105:cofbb]2.0.co;2.
