# Supplementary material for: A case for considering individual variation in diel activity patterns
Source: Behav Ecol. 2017 Sep 11;28(6):1524–31. doi: 10.1093/beheco/arx122 (PMC5873257; doi:10.1093/beheco/arx122)
Supplement: Supplementary Material 2 [file arx122_suppl_supplementary-material2.docx]

**Title:** A case for considering individual variation in diel activity patterns.

**Journal:** Behavioral Ecology

**Authors:** Anne G. Hertel, Jon E. Swenson, Richard Bischof

**Corresponding Author E-mail**: anne.hertel@nmbu.no

**Supplementary material 1**

**Figure 1:**

Sum of squared error scree plots to determine the optimal number of activity-tactic clusters from the placement of activity patterns along the first two PCA axes. The within-group sums of squares ceased to decrease strongly after 4 to 6 clusters, indicating that clustering into more subgroups did not improve the variation explained by the clustering. Activity was observed from movements of brown bears in south-central Sweden in the first three weeks of August.


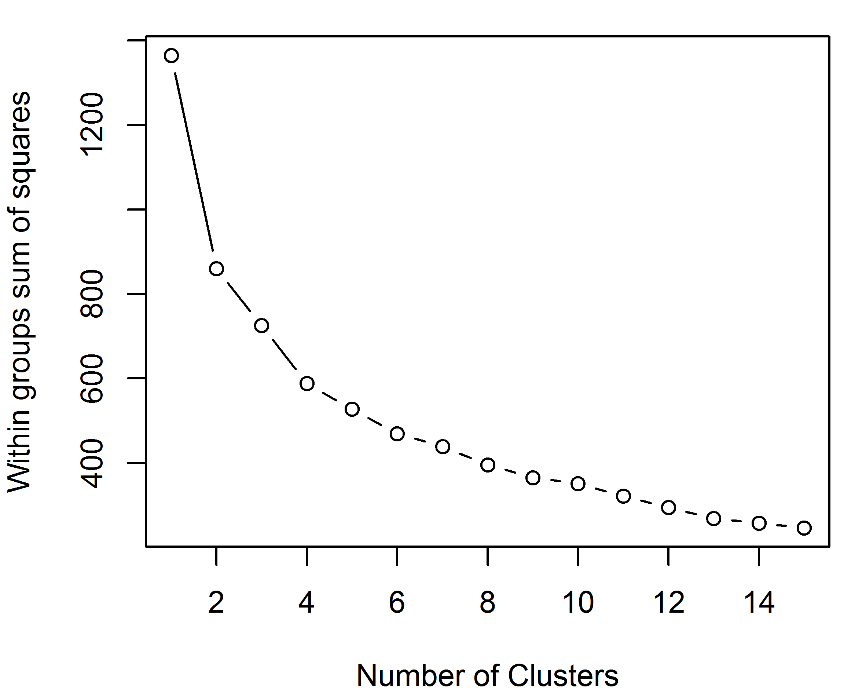


**Figure 2:**

Categorization of brown bear activity patterns into five clusters along the first two axes of the principal component analysis. Extremely diurnal individuals are classified into a separate cluster 5 (orange). Bears in only five bear years out of 196 followed this separate strategy. We therefore decided that four clusters, as presented in the main body of the paper, better represented the diel activity tactics in our population. Activity was observed from movements of brown bears in south-central Sweden in the first three weeks of August.


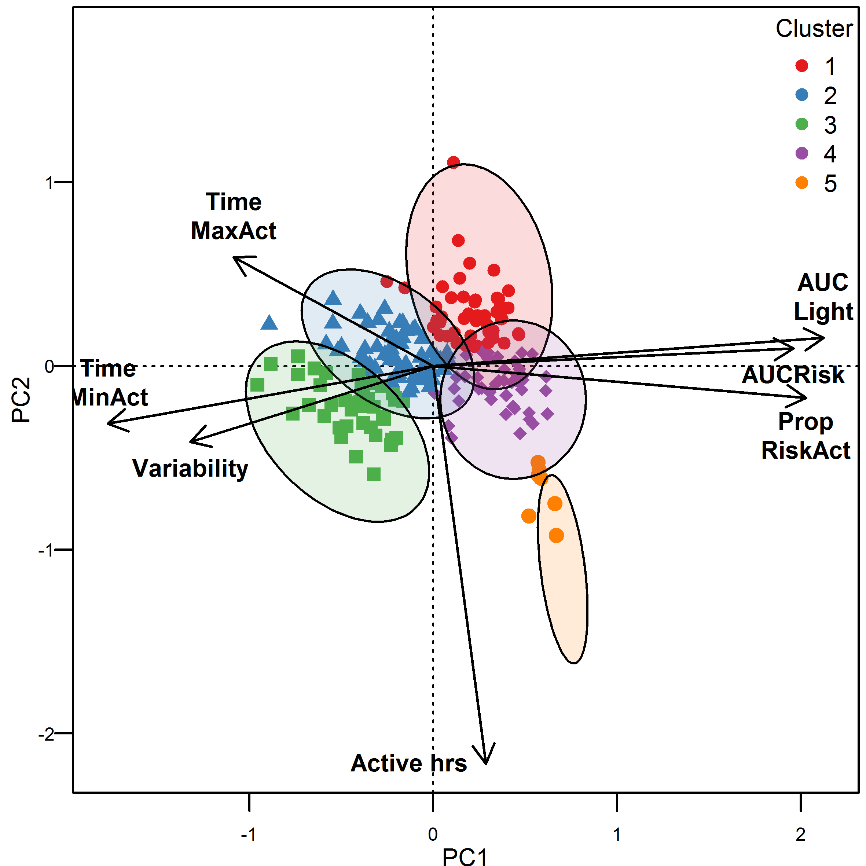


**Figure 3:**

Placement of bear activity patterns along the first three axes of the principal component analysis. Color and character coding is according to the clustering of individuals into distinct activity tactics along the first two PCA axes. Activity was observed from movements of brown bears in south-central Sweden, in the first three weeks of August.

**
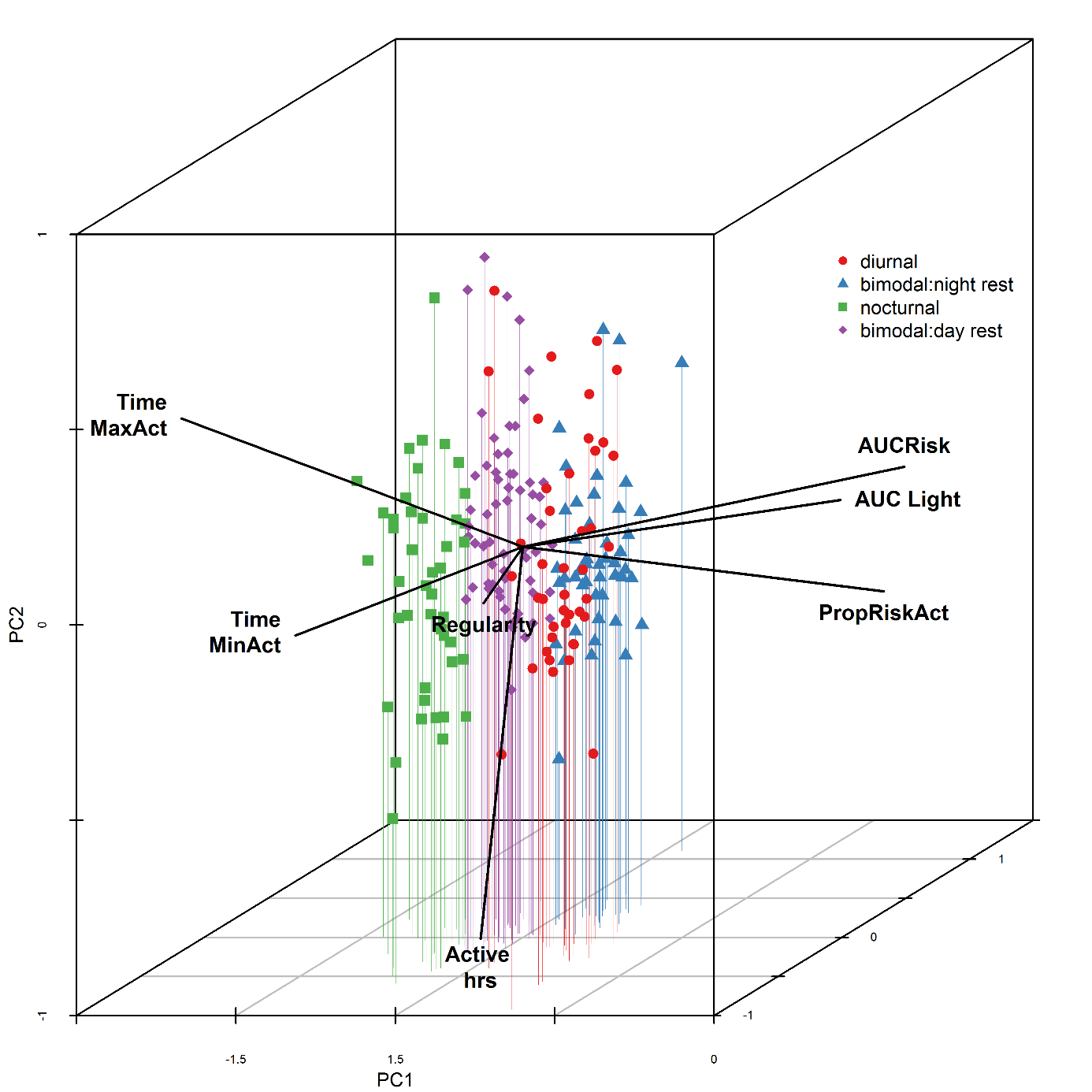
**
